# Exploring the State of Gender-Centered Health Research in the Context of Refugee Resettlement in Canada: A Scoping Review

**DOI:** 10.3390/ijerph17207511

**Published:** 2020-10-15

**Authors:** Chloe Zivot, Cate Dewey, Cole Heasley, Sharada Srinivasan, Matthew Little

**Affiliations:** 1 Department of Population Medicine, University of Guelph, 50 Stone Rd E, Guelph, ON N1G 2W1, Canada; cdewey@uoguelph.ca (C.D.); heasleyc@uoguelph.ca (C.H.); 2 Department of Sociology and Anthropology, University of Guelph, 50 Stone Rd E, Guelph, ON N1G 2W1, Canada; sharada@uoguelph.ca; 3 School of Public Health and Social Policy, University of Victoria, 3800 Finnerty Rd, Victoria, BC V8P 5C2, Canada; matthewlittle@uvic.ca

**Keywords:** women’s health, refugee resettlement, gender, public health, family health, global health, forced migration

## Abstract

Interdisciplinary health research that investigates gender as a relational process is necessary to facilitate a safe and healthy resettlement process for refugees in Canada. This scoping review explores the range, nature, and extent of published research examining gender in relation to refugee health during resettlement in Canada. An initial search of six databases yielded 7325 articles published before June 2019. A total of 34 articles published between 1988 and 2019 were included for in-depth review. Articles meeting inclusion criteria primarily focused on refugee women. Categories of focus included maternal health, social and emotional health, health impacts of sexual and gender-based violence and torture, access to health and social services, decision-making and health-seeking behavior, mental health, and sexual and reproductive health. Our thematic analysis identified connections between gender roles, expectations, ideals, and health through interactions and lived experiences within the family, community, and healthcare system. Review findings suggest that many refugee women are influenced by pervasive gender roles and expectations as well as exposed to gendered health systems and practices that may pose risks to health, particularly mental health and access to services. Further efforts should be made to understand processes and experiences of resilience and community building in countering negative impacts of gendered beliefs and practices on health during resettlement.

## 1. Introduction

Since 2014, Canada has received over 60,000 refugees [1] and remains a preferred resettlement country for many refugees due to the relatively robust supports available to individuals resettled through its Refugee and Humanitarian Resettlement Program [2]. These include pre- and post-arrival healthcare and orientation services, access to loans to cover the costs of travel to Canada, and financial and social support during the first twelve months of resettlement [2]. Often referred to as convention refugees, these individuals or families arrive with pre-determined refugee status and are considered permanent residents upon arrival [2,3]. Incoming refugees often face social and economic barriers and have increased risk of poor physical and mental health outcomes compared to other immigrant classes in Canada [3,4,5]. Refugees often face difficulties finding employment due to relatively lower levels of education, insufficient English language and literacy, limited access to social support services, transportation barriers, unrecognized credentials, and health challenges [6,7,8]. Physical or mental and emotional trauma experienced during flight, asylum, and resettlement may result in decreased capacity to cope with the stressors associated with the necessities of resettlement, such as finding employment and accessing healthcare and social services [9,10]. For some, trauma and pre-existing health conditions may exacerbate, or be exacerbated by, challenges faced during resettlement, resulting in negative mental and physical health outcomes [11].

Women and children currently account for the majority of those who enter Canada with refugee status [12]. In addition to the barriers to health listed above, women often face other unique barriers to health during refugee resettlement that warrant special attention. At present, refugee women resettling in Canada are predominantly of childbearing age [12,13], and as such, often arrive with young children, while pregnant, or experience pregnancy during the resettlement period. Accessing maternal healthcare can pose a significant challenge for newcomer women and families due to structural barriers, including language barriers, unfamiliarity with the Canadian healthcare system and entitlements during resettlement, and challenges with cultural incongruity of care [14,15]. Women face a disproportionate risk of sexual and gender-based violence (SGBV) across the forced migration process, including during resettlement [16]. As a result, refugee women living with human immunodeficiency virus (HIV), those who have experienced female genital cutting, or those who suffer from trauma associated with SGBV may encounter increased social, emotional, structural, and safety-related barriers to health and targeted care services and social supports during resettlement [17,18]. It is also important to recognize that gendered barriers and pathways to healthcare do not only impact women and children. Other populations, and particularly members of lesbian, gay, bisexual, transgender, queer (or questioning), and other sexual or gender minority (LGBTQ+) refugee communities experience barriers to health and healthcare stemming from discrimination related to sexuality and gender.

While it is widely recognized that gender is an important determinant of health, existing literature pertaining to gender and health during refugee resettlement in Canada remains limited. A major criticism of existing refugee health research is its narrow emphasis on quantitative physical and mental health outcomes [19,20,21] and limited integration of refugee voices in conceptualizing and elucidating the barriers and pathways to refugee household health [22,23,24,25,26]. The literature is dominated by research emphasizing biomedical outcomes associated with childbirth (e.g., antenatal care outcomes and maternal depression) as measures of maternal health. However, gender influences health as a relational process characterized by gender roles, expectations, beliefs, ideals, and physical, social, and emotional relations. Viewed in this way, gender is both a critical aspect of one’s identity and a dynamic process of exchange with other individuals and institutions, that often implicitly or explicitly informs experiences and health behaviors [23,24]. As a result, gender actively shapes how health decisions are made as well as who is most vulnerable to negative health outcomes and experiences, and when. As such, it is necessary to understand and characterize the complex pathways through which gender functions as a determinant of health during refugee resettlement.

The primary objective of this review is to outline key pathways through which gender impacts health during refugee resettlement in Canada. Grounded in an ecosystemic conceptual framework [22], this review will examine and characterize the extent and nature of peer-reviewed academic literature examining the relationships between gender and health across different scales—including the micro (family), meso (community), and macro (institutional) realms—among refugees in Canadian communities. Overall, this review aims to identify themes, trends, and gaps in published literature on the gender–health nexus, evaluate methodological trends and gaps in gender-centered refugee health research, and identify opportunities for future research and activities to improve the responsiveness of resettlement organizations and healthcare systems to the complex needs of refugees during and following resettlement in Canada.

## 2. Materials and Methods

Our scoping review was grounded in a methodological framework presented by Arksey and O’Malley [27] and sought to synthesize relevant literature pertaining to gender and refugee health during resettlement in Canada, as well as collective knowledge on gendered pathways and barriers to refugee health during resettlement. Scoping reviews are an effective form of knowledge synthesis when exploring under-researched topics [27], particularly those that engage with a variety of study designs and disciplines. In our case, the scoping review design was appropriate given the interdisciplinary nature of literature investigating connections between gender and refugee health. Methods were conducted in accordance with the standards outlined by the Preferred Reporting Items for Systematic Reviews and Meta-Analyses—Extension for Scoping Reviews (PRISMA—ScR) checklist and guidelines [28]. The guiding question for this review was: what is the range, nature, and extent of peer-reviewed literature examining refugee health during resettlement in Canada from a gendered perspective?

### 2.1. Search Strategy

Articles for review were gathered through a systematic search of six electronic library databases including PubMed/Medline, CINAHL (and CINAHL Plus), Women’s Studies International, PsycINFO, Sociology Collection, and JSTOR. A research librarian was consulted in the design of the review protocol, including in the selection of databases and search terms. The search included all relevant articles published until June 2019. When possible, filters were applied to capture only peer-reviewed academic research articles. Grey literature was not included in this review. Search terms were selected according to the four central components of our guiding question, as outlined in Table 1. Our search strategy also included a hand search, in which four peer-reviewed academic journals were searched for references that may not have been identified by the electronic search. Journals were identified for the hand search according to perceived relevance to the review topic matter, and included: Journal of Refugee Studies, Journal of Immigrant and Minority Health, Ethnicity and Health, and Refuge: Canada’s Journal on Refugees. Literature identified through the hand search was subject to the same inclusion and exclusion criteria as that of the electronic database search.

### 2.2. Study Selection

All articles yielded from the database and hand searches were managed by Mendeley and imported directly from Mendeley into DistillerSR. Duplicates were deleted, and remaining articles underwent screening. All articles were screened independently by the first and second authors (C.Z., C.H.). Screening forms were used for title and abstract screening (Level 1) and full-text screening (Level 2) to enforce inclusion and exclusion criteria. Due to the broad and often subjective nature of the concepts for which we were screening (i.e., gender, health/wellbeing), we developed guidelines for consistent application of inclusion and exclusion criteria (Table 2). The Kappa statistics for level one (title and abstract) and level two (full text) screening were 0.82 (strong agreement) and 0.75 (moderate agreement), respectively [29].

For the purposes of this review, articles were considered to have a “gendered perspective” if they explicitly discussed the health implications of gender roles, norms, stereotypes, identities, ideals, and/or relations, including physical relations such as SGBV. Further, we restricted our target population to individuals and families with legally recognized refugee status in Canada. This decision was made with the understanding that other categories of migrants, and particularly refugee claimants, face a different set of health challenges and legal constraints in Canada than do those with refugee status. Thus, we included research only on refugees with legally recognized refugee status to ensure that we did not conflate the experiences of individuals with secured refugee status in Canada with those of refugee claimants, failed refugee claimants, and undocumented migrants.

### 2.3. Data Management, Extraction, and Analysis

Data charting forms were piloted by both reviewers using the first five included articles by reference identification number. Following finalization of the charting form, data extraction was carried out by the first author (C.Z.). Data charting included extracting data on the name and location of the study, the study population, age, gender, and migration status, study design, methodology including use of community engaged or participatory methodologies, methods, study location, sample size, year of publication, and research aim. We applied a modified version of the mixed methods appraisal tool (MMAT) [30] to assess the quality of each article. Following this, article text was imported into NVivo 12 and for an inductive–deductive thematic analysis, again performed by the first author (C.Z.). The deductive component of the data analysis was informed by an ecosystemic framework [22], during which themes were organized into the micro (family) level, meso (community) level, and macro (institutional level).

## 3. Results

**Figure 1:**
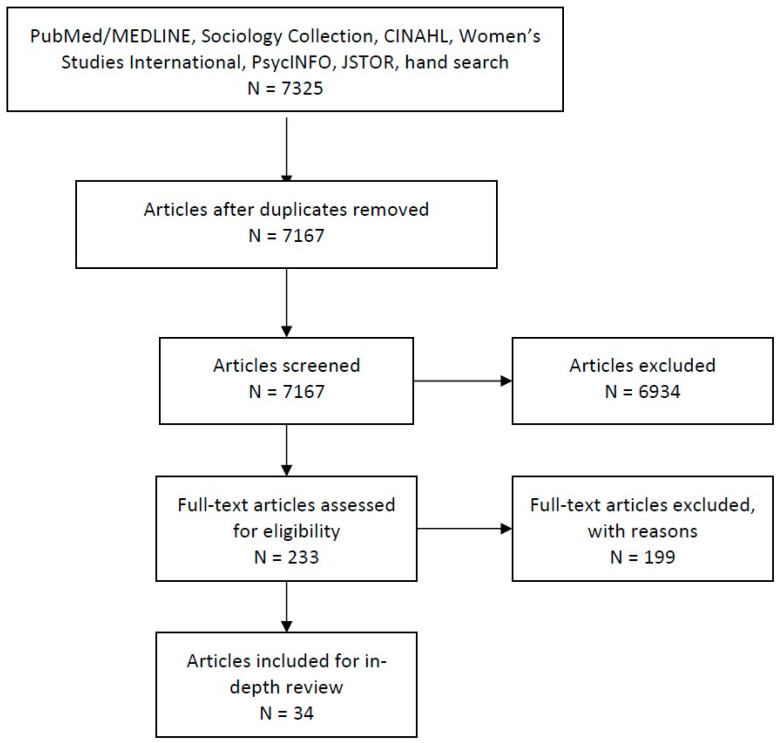
PRISMA chart of article selection.

A total of 34 articles were identified for in-depth review (Figure 1). All of the included articles met the quality assessment criteria according to their respective study design using the MMAT tool [30].

### 3.1. Study Characteristics

#### 3.1.1. Study Design

An overview of study characteristics is presented in Table 3. Of the 34 articles included in our review, 23 (67%) were qualitative, five (15%) were quantitative, four (12%) were mixed methods, and two (6%) were scoping reviews. Common methods included in-depth interviews (13 articles), semi-structured interviews (11 articles), and focus group discussions (nine articles). Other methods used less frequently included surveys, questionnaires, case reports and studies, and participant observation. A community-engaged research approach, which was defined as research that was developed in partnership with a non-academic group or organization, was used in four (12%) of the 34 articles. A participatory action research approach, which was defined as research that involved community partners or research participants in the design, data collection, and analysis stages of the research, was used in two (6%) of the articles. Sample sizes ranged from one to 1536, with the smallest sample sizes occurring in qualitative studies that used ethnographic and case study designs, and the largest sample size occurring in quantitative studies such as prospective cohort and cross-sectional studies. One scoping reviews included 16 published articles and the other included 126 published articles on newcomers.

#### 3.1.2. Study Populations

Publication dates spanned from 1988 to 2019, with the majority of articles published since 2007. Articles reported on studies that were conducted across Canada, with 15 conducted in unspecified locations in Canada, nine in Ontario, five in Alberta, three in Quebec, three in British Columbia, and two in Saskatchewan. Two articles included study populations in more than one province. The majority (22) of articles reported on study populations comprised solely of adult women over the age of 18 years. Only one article included a study population of solely adult men, and three articles included a study population of adult men and women. Two articles focused on youth (ages 13–18 years) and study populations included both young women and young men. Of the 34 articles in our collection, only one article reported on a study population comprised of refugee individuals who identified as LGBTQ+. Three studies incorporated healthcare providers (HCPs) or social service providers in their study population, in addition to refugees. Finally, three articles exclusively included HCPs or social service providers. Over half (19) of the articles included refugees but did not specify pathway through which individuals attained refugee status. Six articles included or discussed government-assisted refugee (GAR) individuals or households. Eight articles included refugees along with other migrant classes but differentiated refugee versus non-refugee specific data in their results. Study populations were from a broad range of geographic and ethnic backgrounds, with main countries of origin including Syria, Sudan, Democratic Republic of Congo, Somalia, Afghanistan, Sri Lanka, and Cambodia.

#### 3.1.3. Article Focus

Given the complexity of refugee health across the migration process and during resettlement, the articles included in our review evaluated and discussed many aspects of the gender–health nexus. Articles were classified into seven primary categories, including: maternal health (eight articles), social and emotional health (seven articles), health impacts of SGBV and torture (five articles), access to health and social services (five articles), decision-making and health-seeking behavior (five articles), mental health (three articles), and sexual and reproductive health (one article). Articles that discussed mental health outcomes within other key categories listed above (for example, in relation to experiences of SGBV or in the antenatal period) were grouped in the overarching category (i.e., SGBV and torture or maternal health instead of mental health).

### 3.2. Results of Thematic Analysis

We used an inductive–deductive thematic analysis approach grounded in an ecosystemic framework, which has been previously proposed for use with immigrant and refugee women in Canada [22]. Accordingly, findings related to interdependent experiences of individual health and gender were organized and summarized in relation to the macro (institutional), meso (community), and micro (family) levels. In our analysis, we have endeavored to accurately represent the range, nature, and extent of refugee health research focusing on gender, including highlighting proportionately the populations referenced by this body of literature. Of all included articles, 31 of 34 either included or exclusively included adult women; as such, this thematic synthesis will primarily describe the experiences of gender and health amongst refugee women during resettlement in Canada. Included articles focusing on youth discussed both males and females and, therefore, while our focus on youth is limited, we too summarize findings related to both sexes within this demographic. Experiences of gender and health amongst refugee populations in Canada cannot and should not be homogenized within or across sub-populations, at the risk of propagating harmful gender and ethnic stereotypes. While summarizing relevant findings, we strive to avoid generalizing relationships between refugee health and gender, which ought to be evaluated in their specific contexts. 

#### 3.2.1. Micro Level: Gender, Health, and the Family

Within the ecosystemic framework, micro-level analysis assesses how individual wellbeing is influenced by the family, including household members as well as extended family at the local, national, and trans-national levels [22].

##### Challenges Fulfilling Gender Roles and Expectations within the Family

Women often expressed that challenges and limitations in their capacity to fulfill gender roles and expectations within the family was a cause of distress during resettlement. Many women emphasized the centrality of motherhood in their own personal identity as well as an important source of hope and self-preservation during resettlement [31,32], and found aspects of resettlement that challenged their conception of what it means to be a good mother particularly distressing [31,33]. Inability to navigate health services and make informed healthcare decisions for their children due to language and other structural and institutional constraints resulted in feelings of powerlessness among some women [34].

The centrality of gender in determining health-seeking behavior was reflected in the way women often de-prioritized their own health, leisure and self-care, and healthcare-seeking in favor of their partners’ and children’s [3,32,35]. Another common experience during resettlement was the inability to care for one’s own health and wellbeing due to childcare roles and responsibilities [32,36,37]. Women found it challenging to find time to access medical services [36] or practice self-care through leisure or physical activity [38] as the absence of extended family limited options for childcare, a responsibility seemingly borne largely by women.

Cultural and language barriers also posed challenges to women’s roles within households, with implications for household health. For example, language barriers and unfamiliarity with Canadian grocery stores and food products also challenged refugee women’s ability to fulfill caregiving roles by presenting barriers to preparing acceptable and healthy foods for their family [33,35]. Changes in children’s food preferences away from traditional foods of their home countries was also a source of household tension [33,35] and increased parents’ difficulty in navigating children’s nutrition, in the face of unfamiliar and sometimes highly processed foods and inability to understand information communicated on food labels. Chulach and colleagues (2016) discussed the central role of breastfeeding in the enactment of motherhood [31]. HIV-positive status complicated this cultural and social expectation for many refugee women, who reported experiences of stigma and judgment resulting from the inability to breastfeed their children and fear of revealing HIV status to family and friends [31].

Refugee men also experienced challenges associated with intra-household gender roles. Simich and colleagues (2010) found that some Sudanese refugee men directly linked their own health with family wellbeing, characterizing their own personal health as the absence of family conflict and perception of being respected by their wives and children [39]. Similar sentiments linking men’s health to family wellbeing were also expressed by Racine and Lu (2015) [40]. Affleck and colleagues found that the inability to meet cultural or perceived expectations as a husband and father resulted in “depleted masculinity”, which participants linked to serious mental health concerns such as depression, isolation, alcoholism, and suicidal ideation, as well as perceptions of heightened incidence and nature of intra-household conflict [41]. In turn, men’s perceptions of failure to meet gender expectations may impact the mental or physical wellbeing of other household members as well as economic and social resettlement outcomes more broadly.

Several articles described how household decision-making power and control may affect the health of family members [36,42]. In some refugee households, decisions about reproductive health and contraception practices were controlled by men [42,43], sometimes resulting in unintended or unwanted pregnancies among their partners. According to women, male partners often expressed little to no interest in child immunization decisions [44], suggesting that gender-based decision-making power is not experienced or exerted uniformly across family health decisions.

##### Changes in Gender Roles and Expectations and Family Dynamics

In addition to distress caused by challenges to traditional gender roles, changes in gender roles and expectations and family dynamics acted as a stressor for refugee families during resettlement [39,45,46]. Underemployment, inability to secure employment, discrimination or harassment in the workplace, or the potentially new expectation to work outside the home were cited as major stressors during resettlement, particularly when accompanied by changes in intra-household gender relations and distribution of labor [39,41]. In circumstances where women worked outside the home for the first time, re-negotiating the division of household responsibilities (e.g., preparing meals and caring for children) was challenging and occasionally resulted in marital conflict. Yet, in some instances, there was also willingness to embrace changing gender roles during resettlement, with both women and men recognizing that adaptations to marital roles were required in light of a new social and economic environment [39]. More generally, HCPs noted that supporting refugees, particularly single female-headed households or those impacted by trauma, in accomplishing necessary tasks such as finding housing, employment, and arranging children’s or adult education, was often a necessary pre-cursor to addressing psychological distress through traditional means of therapy [32].

Men and women both reported difficulty in managing household relations and barriers to mitigating marital conflict in the absence of support from extended family members [39,47]. Whereas many men turned to male relatives for advice related to marriage and childrearing in pre-migration contexts, such supports were limited in Canada, resulting in uncertainty and sometimes conflict escalation during resettlement [39,47]. When investigating Sudanese refugees’ conceptions of home and family life in relation to mental health, Simich and colleagues (2010) found some men deeply feared the police becoming involved in family matters and reported hearing of instances when these concerns would contribute to household disbandment, whereby men would leave the family home, sometimes returning to their home countries while their wives and children remained in Canada [39]. Fears around law enforcement involvement and resulting family disbandment may result in reluctance to seek support for managing intrahousehold conflict or other aspects of wellbeing.

Within this navigation and restructuring of family roles, identities, and expectations in the home, children and youth may also be impacted and struggle with resettlement and acculturation. Two articles examined family environment and history of family trauma (respectively) in relation to emotional and behavioral symptoms in refugee adolescents. Rousseau and colleagues (2004) [48] suggested that family environment was associated with behavioral symptoms such as hyperactivity, opposition, and feelings of competency in adolescents [48]. While having a two-parent household may be a protective factor against internalizing symptoms (such as anxiety and depression), adolescents from single parent households reported greater feelings of competency [48]. Intra-household conflict may be compounded by the presence of adolescents; indeed, parents of adolescent girls reported an increase in conflict from baseline (age 13–14) to follow-up (age 15–16). This may relate to the increased pressure girls face in balancing social impacts of acculturation with parents’ desire for stricter adherence to cultural norms, gender expectations, and ideals from their home country [48]. For example, Bokore (2013) reported on women’s concerns with raising daughters in Canada’s liberal culture around dating and premarital sex [49]. Mothers often worried their daughters would become pregnant while unmarried and bring shame and hardship upon themselves and their family. Rousseau et al. (1999) found that family trauma may protect against externalized symptoms (such as aggression), risk behaviors, and school failure in boys and foster positive social adjustment in girls [50]. This may stem from the expectations of parents who, after having endured hardship during migration and resettlement, tend to exert pressure on their children with the hope they will succeed (beginning with academic success) in Canada [50].

#### 3.2.2. Meso Level: Gender, Social Support, and Community Building

Meso-level analysis focuses on the influence of community (comprised of informal and formal social networks) on individual health [22]. Resettled refugees in Canada frequently reported feelings of isolation, driven by limited social support and diminished sense of community. Conversely, social support and sense of community were identified as important in facilitating health and coping with mental illness during resettlement [36].

##### Loss of Social Support Networks and Sense of Belonging

Limited social support was often cited as an acute challenge during child-rearing, including pregnancy, the antenatal period, and childcare. In general, women struggled to engage in social or leisure activities during resettlement for a number of reasons. Female refugee participants often contended with reduced familial and social support in Canada compared to their countries of origin, where they depended on extended family to assist with attending appointments and provide childcare as well as advice and comfort [3,44]. Women also experienced increased difficulty in accessing health information during resettlement, as social networks were often a primary vehicle for health promotion information in their home countries [3,44]. In qualitative interviews, participants commonly noted having to adapt to the different social norms in Canada, where it is less common to drop by unannounced for a visit with friends and neighbors [36,38]. This seemingly impacted women more acutely if women were single heads of household or where male partners worked outside of the home while women primarily remained alone or with children inside the home [34]. Without the support of extended family and social networks, women often struggled to balance their roles as primary caregivers to children with important leisure activities such as physical activity and participation in religious and spiritual events [36,38]. These findings are important since isolation and poor social support are associated with poorer health outcomes among refugees. Spiritual and religious activities are a protective factor against mental illness and facilitated adaptation to resettlement community [36,38,51].

Evidence suggests that women experienced the most isolation during the post-partum period, which heightened risk of post-partum depression (PPD) [31,52,53,54]. Further risk factors for mental illness, PPD in particular, included low income, food insecurity, low levels of education, concern for family left behind in war zones [39], and prior experience of abuse [54]. Overall, Dennis and colleagues (2017) found that migrant women had significantly higher rates of depressive symptoms at 16 weeks post-partum than their Canadian-born counterparts [54].

Men also experienced impacts of decreased social support from family members and friends, as well as from a lack of sense of belonging and worthiness in Canadian society. Beiser and Hou (2017) examined predictors of positive mental health among refugees using results from Canada’s General Social Survey and found that a sense of belonging in Canada was a significant predictor of mental health amongst refugee men [55]. This study also found that self-reported discrimination was associated with poorer health outcomes among refugee men, although not among women despite women reporting a higher number of discriminatory events [55].

##### Navigating Social and Cultural Environments

Beliefs and practices within one’s ethnic community and social network play an important role in health-seeking behavior and access to information pertaining to health [56]. Social networks are an important source of information during initial resettlement in Canada. Specifically, refugees often look for advice from others in their resettlement community when making healthcare decisions for themselves and their children. In instances when traditional health practices and beliefs contradict or are prioritized over those of the Canadian healthcare system, some women may refrain from accessing health services or feel distress while navigating health decisions that contradict the beliefs of those of other members in their cultural or resettlement communities [35,45,56,57].

A widely reported theme was the complexity of navigating gendered and stigmatized health challenges while building social networks within resettlement communities. For example, refugees struggling with mental health, those living with HIV, or survivors of sexual violence revealed complex social challenges adapting to resettlement. Refugee women may feel a desire or pressure to conceal experiences of sexual violence or HIV status from family and community members. This was particularly true for women from communities where patriarchal values and beliefs necessitate women be perceived as “pure” in order to command respect, conform with gender expectations, and maintain marital relationships [32,49,58]. In such instances, women feared that if their experiences and status as a survivor of sexual violence were exposed, they would lose their marriage, community, and social support networks [32,49]. In some instances, women who were survivors of sexual violence during conflict or migration reported fears that their husbands would feel obligated to leave them if their victimization was to become known in the extended family or community, due to pervasive social norms that continued to exist during resettlement [49]. Women living with HIV had similar fears, and in qualitative interviews, some women reported losing their social networks or relocating to a different community after their HIV-positive status was publicized [31]. Chulach and colleagues (2016) discussed challenges associated with pregnancy and childbirth during resettlement among HIV-positive refugee women in Ottawa [31]. Participants experienced stress when having to conceal their HIV-positive status while participating in social activities while pregnant or post-partum, particularly in fielding questions around breastfeeding, or their lack thereof [31]. During pregnancy, women also found it difficult to find privacy to take their daily medication [31]. Many participants reported silence and stigma around mental health, HIV, and history of sexual violence [31,32,49]. In some circumstances, associated fear of having related occurrences exposed within their community resulted in distrust between community members, inhibiting the development of friendships and exacerbating refugees’ sense of isolation during the resettlement period [31].

#### 3.2.3. Macro Level: Gender and Health-Seeking Behavior, Access to Health and Health Services

Macro-level analysis considers how health, educational, social, and economic policy environments, institutions, and systems work together to influence individual wellbeing [22]. Many articles reported on the gendered dimensions of refugees’ experiences with health institutions and services. Structural barriers grounded in gender, as well as cultural incongruity, discrimination and lack of sensitivity, confidentiality concerns, and shame associated with gender beliefs and ideals established barriers to seeking care during resettlement.

##### Structural Barriers to Healthcare

Language barriers were a key structural factor that prevented refugees from seeking appropriate care [3,34,36]. Professional language interpretation services are often not provided by HCPs during medical appointments [59]. Language barriers disproportionately impact women, who are often slower to learn Canadian official languages than male partners and children due to their lower exposure to settings outside the home [33]. As a result, women are often required to take their children, partners, or family members along with them to medical appointments to act as translators [3,36]. This practice may affect consent, confidentiality, and transparency, particularly when seeking services or information for issues may be personal or stigmatized such as sexual and reproductive health, sexual or gender-based violence, or mental health [3,34,56]. When working with Karen refugees (an ethnic group from Myanmar), Clark (2018) found that even when professional interpreters were available to women in their resettlement communities, some were not comfortable arranging for their services, or were hesitant to disclose personal information, particularly if there was a discrepancy in gender, ethnic background, or sexual orientation between the interpreter and the patient [37]. Participants in a study by Donelly and colleagues (2011) consistently identified the need for translated materials on mental health and other health services as a means to improve access to healthcare [36]. Culturally sensitive health awareness and education campaigns were identified as another potential pathway to improved access to health services as well as overall health [36,58].

Transportation to and from medical appointments was often cited as a barrier to healthcare [34,42], and disproportionately impacted refugee women who, due to language, social, and gendered cultural constraints, may be uncomfortable, unwilling, or unable to travel alone to attend medical appointments [42]. Barriers to healthcare that impact women may also impact refugee children’s health [57], especially in instances where women are the primary caregiver of children [37]. 

##### Cultural Incongruity and Lack of Sensitivity

Cultural incongruity poses another major obstacle to access and utilization of health services, particularly during pregnancy and childbirth. Several articles discussed the incongruity women faced between Western medical practices and their own personal and cultural beliefs around childbearing [43,45,56]. Higginbottom and colleagues (2013) discussed the belief amongst Sudanese women of childbirth as a “natural” process [45]. There were several implications of this belief: for example, women and families may feel that birth in an institutional setting or taking medication for pain relief are unnecessary or unnatural. Further, there is the gender expectation in some communities that women remain strong or stoic during childbirth [45], resulting in women hiding discomfort and preventing HCPs from administering pain relief. The possibility of caesarean section surgeries [31,60] or being assigned a male physician for delivery [60] contributed to some women’s hesitation to give birth in a hospital setting [45]. Some women who were not accustomed to giving birth in a supine position reported increased discomfort during childbirth in Canada [45]. Finally, some cultural norms of infant breastfeeding and pre- and post-natal food consumption were not met in hospital settings [45], with potential implications for maternal nutrition or willingness to prolong the hospital stay when required.

Refugee populations and healthcare providers described the role that discrimination and poor cultural sensitivity can play in limiting access to healthcare [36,61]. Some refugee women described feeling shame as a result of hostility, condescension, racism, and judgment while accessing health services [46,57,61]. Some women who were HIV-positive experienced discriminatory or insensitive comments from healthcare providers while seeking healthcare during pregnancy, which reduced perceived quality of care and produced feelings of shame [31]. Specifically, lack of sensitivity and availability of appropriate service providers was reported in reference to refugees’ access to mental health care services [36,62]. For example, in an instance recorded by Donelly and colleagues (2011), after raising concerns about her mental health, a woman was advised by her general practitioner to make herself feel “good” by purchasing new clothes, getting her hair done, and instructing her husband to give her jewelry and money to spend on luxury items [36]. Participants in Kahn and colleagues (2018) reported that mental health support for LGBTQ+ refugees is often beneficial and recommended by resettlement workers and other gateway providers; however, it is often difficult to find an appropriate mental health provider who can exhibit sensitivity and empathy toward the experiences of clients navigating dual minority identities as LGBTQ+ and forced migrants [62]. While matching provider with patient according to similar backgrounds and characteristics may ameliorate some of the challenges to providing quality care during resettlement [62], HCPs would benefit from further training in cultural and gender competency in care [58]. Experiences of impatience from HCPs and administrative staff were commonly reported by refugees, resulting in some hesitation—particularly among women—to seek healthcare unless urgently required. Furthermore, refugees reported experiencing institutionalized discrimination, whereby healthcare services are refused to them based on confusion surrounding insurance coverage, or due to HCPs perception of hassle or uncertain reimbursement associated with treating refugees for whom certain services or covered only by the Interim Federal Health Program [57,61].

##### Confidentiality when Accessing Healthcare

Concerns around confidentiality reportedly impacted health-seeking behavior, particularly for health outcomes considered by refugees to be stigmatized in their families and communities (e.g., post-traumatic stress disorder, depression, anxiety, and SGBV and torture) [63]. Gender expectations regarding the performance of motherhood, in which women are expected to exhibit strength and avoid behavior that may be interpreted as weakness, may prevent some women from accessing mental health services to treat maternal or post-partum depression [45]. The prospect of being identified as someone experiencing mental health challenges or as the victim of sexual violence, as well as the fear of unknown repercussions of this label, prevented refugees (particularly women) from seeking related health services [36]. Refugees feared being recognized by community members at mental health or trauma counseling appointments. They also feared being faced with an interpreter with unknown connections to their communities in Canada or their country of origin who may expose their history of mental illness of sexual violence to family and community members, which may result in loss of social status, isolation, and conflict [36]. This same fear was echoed by LGBTQ+ forced migrants [62]. Furthermore, in some circumstances, shame associated with perceived loss of purity prevented women from discussing their experiences at all [49], many preferring to try to forget about these traumatic experiences as a way to cope [32,58]. Finally, there were concerns surrounding interactions with the healthcare provider themselves; some women feared discrimination and stigmatization from HCPs [36] and consequent feelings of shame. Some also worried that if they reported particular health concerns to the HCP, their information could be used against them [46], with a common fear being that state actors would remove their children from their care [36,57]. Others reported general distrust of HCPs, and some expressed fear of experiencing sexual violence at the hands of their HCP (both within and outside the context of mental health and trauma care-seeking) [60]. In turn, Berman and colleagues (2009) reported on two study participants having experienced sexual violence from HCPs during resettlement in Canada [46].

## 4. Discussion

To our knowledge, this is the first review to collate, analyze, and summarize research on the gender–health nexus among refugee populations in the Canadian context, framing gender as an active and relational process that shapes experiences of health and wellbeing. We aimed to identify relevant themes with the gender–health nexus in refugee literature in Canada, as well as methodological trends, gaps, and opportunities for future research. We also sought to look to the literature to identify opportunities and activities for service providers and other stakeholders to improve the responsiveness of the Canadian healthcare system to the complex needs of refugees during and following resettlement in Canadian communities.

Gender roles, expectations, ideals, and relations as enacted at the micro, meso, and macro levels impact health and access to healthcare during refugee resettlement in Canada. By examining gender as both a nuanced component of one’s identity as well as a dynamic process that is experienced in relation to other people and institutions, we can better understand how health decisions are made and who is most vulnerable to negative health outcomes and experiences during the resettlement period. Furthermore, in a broader sense, using a gendered perspective to investigate health in multi- or cross-cultural contexts may allow us to look beyond individualism prominent within Western healthcare models to consider the heterogeneity of conceptualizations of health across ethnic communities and cultures, genders, and social environments. Doing so may catalyze more relevant and valid research and knowledge production leading to inclusive and respectful care by healthcare systems.

Our review suggests that gendered experiences of isolation, language barriers, sociocultural environment of the resettlement community, family roles and expectations, and access to healthcare services and supports intersect to produce and perpetuate gender-based health inequities for newcomers to Canada, in particular for women from refugee backgrounds. Two major themes identified in our review were: (i) experiences and perceptions of a lack of agency or control in daily life and health-seeking; and (ii) limited social support during the resettlement process. Both of these have the potential to negatively impact health and wellbeing of refugee populations. Therefore, our findings suggest the importance of committing time and resources toward research that investigates pathways and barriers to agency, social support, and community building during the resettlement.

Further opportunities may exist to leverage public and community health initiatives and mental health therapy as pathways to social support and community building. Support groups, community health initiatives, and immersive social programming that aim to bring community members together to support and learn from one another may contribute to improved health and wellness of refugee individuals, families, and communities [58]. In order to strengthen support for this shift from individual to community-centered health and social programming, research should be conducted to evaluate the impacts of these initiative where possible. Yet, as highlighted in our results, it is crucial that these programs integrate and mitigate concerns around confidentiality, cultural sensitivity and safety, and language and structural barriers. As such, it is critical that researchers partner with community and resettlement organizations and refugees themselves to design and facilitate culturally sensitive research that accounts for social and cultural norms and participants’ migration histories in order to produce nuanced, context-specific knowledge that is mobilized to produce positive impacts on the health of newcomers. Participatory action research can be particularly effective in this regard, as demonstrated by relevant articles in our review, as well as by others who have successfully used this approach with refugees in other research and geographical contexts [64,65,66]. A participatory approach can support the collection of more situationally accurate and nuanced data, while also building research capacity and helping to ensure that social and cultural challenges are navigated appropriately in order to protect the safety and wellbeing of all those involved.

Our review highlights several gaps in the literature and opportunities for future research. Literature from our review highlights that few articles examine resilience strategies for refugee women to overcome challenges they face during resettlement [34,51]. Strength-based research focused on understanding the ways in which women cope with and overcome structural, environmental, and social challenges during resettlement may promote improved supports for refugees, which in turn may improve wellbeing of incoming refugees to Canada. Evidence from our review suggests that research that investigates health on a per individual basis may not fully illuminate the complex determinants of health and needs of refugees, particularly those with children. Therefore, further research is required to better understand how the dynamism of gender roles, norms, and expectations during resettlement in Canada impact the mental health and access to services of various members of refugee households, which further frames the family as the unit of analysis. Furthermore, future research should be conducted to expand the body of literature which seeks to illuminate the pathways through which men experience gender and health during resettlement, and the implications on household wellbeing via their role as partners and fathers.

There is a need for a systematic review on gender and health of migrant groups including but not limited to those with refugee status. During our review process, articles were often excluded on the basis of the migrant class or migration status of study populations, which often included but did not differentiate economic and family class immigrants, refugees, and refugee claimants. While scholars have advocated for population-specific research when investigating the health of newcomers to Canada, it is important to identify and seek to understand how experiences of gender and health may be different according to migration pathway and status. Conducting a review that includes all migrant classes and compares gender processes and outcomes across populations may provide evidence for interventions with a high degree of specificity. Particularly, our inclusion criteria did not result in inclusion of many articles pertaining to the health of LGBTQ+ newcomers. Therefore, a scoping review focused specifically on the relationships between gender and health in LGBTQ+ refugee (including claimant) populations in Canada would be a substantial contribution to the research landscape. Additionally, the broader refugee health research landscape may further benefit from a review of similar nature to this one, that synthesizes the state of gender-centered refugee health research in other resettlement contexts, such as resettlement countries in the United Kingdom and Europe.

This scoping review is subject to several limitations. First, design restrictions limiting the review to only articles including study populations with refugee status in Canada resulted widely in the exclusion of LGBTQ+ forced migrants. This is seemingly due to the fact that the majority of articles investigating the health of sexual minority migrants during the initial resettlement period focus on interactions between gender, sexual orientation, and health during the refugee claimant process, before individuals receive official refugee status. By including only articles that report on populations with refugee status, we are unable to add substantially to collective knowledge around this vulnerable population who are deeply impacted by gender expectations and related discrimination. A second and related limitation of our review is that despite exclusion criteria, inconsistent application of the term refugee in existing literature may have resulted in the inclusion of articles with study populations who may be considered refugees by definition, but do not have official legal refugee status in Canada.

## 5. Conclusions

As our review clearly demonstrates, gender is an important determinant of refugee health during resettlement in Canada and requires attention and consideration when designing future research and health initiatives with refugee populations, particularly with refugee women. Gendered experiences and beliefs seemingly exacerbate challenges and barriers to health faced during resettlement, primarily amongst women, leading to increased barriers to health. Meanwhile, perceived threats to or changes in gender roles and family dynamics during resettlement may in some instances cause distress, impacting physical and emotional health and access to resources and services. Social isolation and decreased sense of community may in some circumstances exacerbate challenges encountered during refugee resettlement. At the same time, the perceived necessity to conceal certain experiences or health conditions can be stressful for refugee women and may lead to distrust and stand in the way of community building. It is our hope that this scoping review contributes to a shift in the conceptualization of refugee health and related research priorities to emphasize the importance of lived experiences and social determinants of health, in addition to biomedical health outcomes. By outlining the range, nature, and extent of research incorporating and prioritizing gender considerations related to refugee health during resettlement in Canada, we hope to inform future research, policy, and community-based public health and healthcare programming that seeks to improve refugee health from a holistic and contextualized perspective.

## Figures and Tables

**Table 1:** Search terms.

**Refugee**	refugee* OR forced migrant* OR forced migration
**Health**	health OR healthy OR wellbeing OR well-being OR well being OR wellness OR illness OR disease OR chronic OR disorder OR healthcare OR health services OR access to health OR quality of life
**Gender**	gender* OR woman OR women OR female OR mother* OR wife OR man OR men OR male OR father OR husband OR maternal OR paternal OR family OR families OR sex OR gender relations OR girl* OR boy* OR partner OR marriage OR married OR sex roles OR gender roles OR gender identity OR transgender OR spouse OR interpersonal relations
**Canada**	Canada OR Canadian OR Ontario OR Quebec OR British Columbia OR Nova Scotia OR Saskatchewan OR Alberta OR Manitoba OR Newfoundland and Labrador OR Newfoundland OR Prince Edward Island OR New Brunswick OR Yukon OR Northwest Territories OR Nunavut OR Toronto OR Vancouver OR Montreal

**Table 2:** Inclusion/exclusion criteria for levels 1 and 2 screening.

Level	Inclusion Criteria	Exclusion Criteria
1. Title and abstract screening	Mentions refugees or migrants (unspecified)	Asylum-seekers (refugee claimant), immigrants, or other non-refugee migrant classes
	Mentions one of the search terms for gender (exception of “sex”)	
	Any aspect of physical, mental, or emotional health	Sociocultural aspects or social determinants of health not clearly linked to health outcomes or access
	Study population in Canada	
	Academic journal article	
2. Full text screening	Article including or pertaining to those with refugee status	Articles that included individuals/ households with refugee status but aggregated data or did not specify status
	Articles that examine (relational) aspects of gender: roles, expectations, beliefs, relations	Articles that used gender as a variable, focus on one gender but do not explore relations
	Focus of article is any aspect of physical, mental, or emotional health or a social determinant of health	
	Study Population is in Canada	Articles that included but were not limited to a study population from Canada, but did not specify resettlement country of participants in analysis
	Peer-reviewed academic journal article available in English	

**Table 3:** Overview of article characteristics.

Author(s) Year	Study Population: Migrant Status and Gender	Study Design/Methodology	Objective of Article
**Maternal Health**			
Ahmed, Bowen, Xin Feng 2017	Refugees from Syria (status unspecified) Women	Mixed methods: focus groups, questionnaires	Understand and characterize maternal depression among Syrian refugee women
Brown-Bowers et al., 2015	Refugees (status unspecified) Women	Theoretical	Describe experiences of postpartum depression in women from a critical psychology perspective/socioecological framework
Chulach, Gagnon, Holmes 2016	Refugees who were HIV-positive (status unspecified) Women	Qualitative: semi-structured interviews	Describe and explore meaning and experience of pregnancy from the perspective of HIV-positive refugee women
Dennis, Merry, Gagnon 2017	Refugees and other migrant classes with separated data Women	Quantitative: questionnaires	Identify risk factors for postpartum depression across migrant classes
Higginbottom 2013	Refugees (status unspecified) Women	Qualitative: focus groups	Investigate beliefs around pregnancy and childbirth and discuss relationship between cultural/personal beliefs and maternity services
Khanlou et al., 2017	Refugees and other migrant classes with separated data Women	Scoping review	Review on maternal health among immigrant and refugee women in Canada
Kulig 1989	Refugees (status unspecified) Women	Qualitative: in-depth interviews, participant observation	Describe women’s cultural knowledge of conception and fetal development and the impact on birth control and prenatal care
Winn, Hetherington, Tough 2018	Healthcare providers Women	Qualitative: in-depth and semi-structured interviews	Understand the experiences of healthcare providers of pregnant refugee women
**Social and Emotional Health**			
Chung, Hong, Newbold 2013	Refugees (status unspecified) Women	Qualitative: in-depth and semi-structured interviews	Examine how resilience is promoted, reinforced, or grown among low-income single refugee women
Dyck, Dossa 2007	Refugees and other migrant classes with separated data Women	Qualitative: focus groups, semi-structured interviews	Describe practices and means through which women facilitate and create “healthy space”, as they pursue individual and family health in Canada
Edge, Newbold 2013	Refugees and other migrant classes with separated data All genders	Scoping review	Describe existing knowledge on experiences of discrimination by newcomers and impact/relation with health and access to health services
Hurly 2019	Refugees (status unspecified) Women	Qualitative: semi-structured interviews	Explore definitions of leisure and sources or practices of leisure of three resettled African refugee women
Racine, Lu 2015	Refugees (status unspecified) All genders	Qualitative: in-depth interviews	Describe experiences of multiple forced displacements and resettlement in a mid-sized city
Rousseau, Drapeau, Platt 2004	Refugees or refugee backgrounds from Cambodia (status unspecified) Youth All genders	Quantitative: in-depth interviews	Examine family environment and acculturation over adolescence and their emotional impacts on young Cambodian boys and girls
Rousseau, Drapeau, Platt 1999	Refugees or refugee backgrounds from Cambodia (status unspecified) Youth All genders	Quantitative: questionnaires	Investigate the effect of war-related trauma on subsequent social adjustment and functioning of adolescent Cambodians with refugee background
**Health Impacts of Sexual and Gender-Based Violence and Torture**			
Berman, Giron, Marroquin 2009	Refugees (status unspecified) Women	Qualitative: in-depth interviews, focus groups	Describe experiences of refugee women who have experienced violence in context of war
Bokore 2013	Government Assisted Refugees from Somalia Women	Qualitative: case studies	Describe pre- and post-migration experiences of Somali refugee women, particularly in relation to impacts of sexual and gender-based violence and trauma on health and wellbeing during resettlement
Fornazzari, Freire 1990	Refugees (status unspecified) Women	Quantitative: case reports	Compare demographic information, psychological effects of torture and recovery rates of women who have experienced direct versus indirect psychological or physical torture
Moussa 1998	Refugees (status unspecified) Women	Theoretical	Describe violence against women and gender oppression more broadly in the context of refugee crisis as well as in Canadian resettlement
Yohani, Hagen 2010	Healthcare providers (Women)	Qualitative: case study	Institutional case study describing the author’s (healthcare provider) experience with facilitating a healing program for refugee women survivors of war-related sexual violence during resettlement, both challenges and impacts.
**Access to Health and Social Services**			
Clark 2018	Government Assisted Refugees Women Healthcare and social service providers	Qualitative: in-depth and semi-structured interviews, focus groups, participant observation	Describe Karen refugees’ experience with community support and factors impacting mental health during resettlement
Floyd, Sakellariou 2017	Government Assisted Refugees from SSA Women	Qualitative: semi-structured interviews	Describe experiences of accessing healthcare of non-literate, non-English speaking women from sub-Saharan Africa (SSA)
Guruge et al., 2018	Government Assisted Refugees from Syria Women	Qualitative: focus groups	Describe healthcare needs and experiences of recently resettled Syrian women
Kahn et al., 2018	Refugees Healthcare and social service providers All genders	Qualitative: in-depth and semi-structured interviews	Describe pathways and barriers to mental health care for LGBTQ+ forced migrants
Redwood-Campbell et al., 2008	Refugees from Kosovo Women Sponsors of Kosovar Refugees All genders	Mixed methods: questionnaires, focus groups	Describe Kosovar women’s unmet healthcare needs and barriers to accessing healthcare according to sponsors
**Decision-Making and Health-Seeking Behavior**			
Donnelly et al., 2011	Refugees and other migrant classes with separated data Women	Qualitative: in-depth interviews	Describe supports and barriers refugee and immigrant women face to accessing mental healthcare experiences services of refugee and immigrant women
Kowal, Jardine, Bubela 2015	Refugees and other migrant classes with separated data Women	Qualitative: semi-structured interviews	Describe information-gathering and decision-making about self and child vaccine acceptance and uptake by migrant mothers
Mannion, Raffin-Bouchal, Henshaw 2014	Government Assisted Refugees Women Healthcare providers	Qualitative: in-depth interviews, focus groups, participant observation	Evaluate acceptability of the “Market Guide” created by authors to assist refugees in grocery shopping during resettlement. Describe barriers to buying nutritional foods during resettlement
Wahoush 2009	Refugees and refugee claimants with data separated Women	Mixed methods: focus groups, semi-structured interviews	Describe health-seeking behaviors of refugee mothers responding to an ill preschooler
Yohani, Okeke-Ihejirika 2018	Healthcare providers	Qualitative: semi-structured interviews	Describe experiences and help-seeking behaviors of African refugee survivors of sexual and gender-based violence, from perspective of mental healthcare providers
**Mental Health**			
Affleck et al., 2018	Refugees (status unspecified) Men	Qualitative: in-depth interviews	Describe impacts of war, migration, and resettlement on mental health, specifically in relation to conceptions of masculinity
Beiser, Hou 2017	Refugees and other migrant classes with separated data All genders	Quantitative: computer-assisted telephone interviews	Determine whether refugees have lower levels of positive mental health than economic and family class immigrants from the same source country, and if so what experiences contribute to this disadvantage and to what extent does gender modify the relationships between mental health and post-migration experience
Simich, Este, Hamilton 2010	Refugees (unspecified)-Predominantly Government Assisted Refugees All genders	Mixed methods: in-depth interviews, surveys	Describe family and social factors that impact mental health during resettlement through conceptions of the meaning of “home”
**Sexual and Reproductive Health**			
Kulig 1988	Refugees (unspecified) Women	Qualitative: in-depth interviews, participant observation	Describe relationship between traditional knowledge around conception and the use of birth control Study population included one male traditional healer
